# Memory-Enhancing Effects of the Crude Extract of *Polygala tenuifolia* on Aged Mice

**DOI:** 10.1155/2014/392324

**Published:** 2014-03-12

**Authors:** Zongyang Li, Yamin Liu, Liwei Wang, Xinmin Liu, Qi Chang, Zhi Guo, Yonghong Liao, Ruile Pan, Tai-Ping Fan

**Affiliations:** ^1^Institute of Medicinal Plant Development, Chinese Academy of Medical Science, Peking Union Medical College, Beijing 100193, China; ^2^Department of Pharmacology, University of Cambridge, Cambridge CB2 1PD, UK

## Abstract

Learning and memory disorders arise from distinct age-associated processes, and aging animals are often used as a model of memory impairment. The root of *Polygala tenuifolia* has been commonly used in some Asian countries as memory enhancer and its memory improvement has been reported in various animal models. However, there is less research to verify its effect on memory functions in aged animals. Herein, the memory-enhancing effects of the crude extract of *Polygala tenuifolia* (EPT) on normal aged mice were assessed by Morris water maze (MWM) and step-down passive avoidance tests. In MWM tests, the impaired spatial memory of the aged mice was partly reversed by EPT (100 and 200 mg/kg; *P* < 0.05) as compared with the aged control mice. In step-down tests, the nonspatial memory of the aged mice was improved by EPT (100 and 200 mg/kg; *P* < 0.05). Additionally, EPT could increase superoxide dismutase (SOD) and catalase (CAT) activities, inhibit monoamine oxidase (MAO) and acetyl cholinesterase (AChE) activities, and decrease the levels of malondialdehyde (MDA) in the brain tissue of the aged mice. The results showed that EPT improved memory functions of the aged mice probably via its antioxidant properties and via decreasing the activities of MAO and AChE.

## 1. Introduction

As a result of increased human life expectancy, age-related learning and memory disorders have become prevalent in the aging population, even in the absence of neurodegenerative diseases such as Alzheimer's and Parkinson's disease. To tackle such a major global healthcare issue, it is vital to develop effective prophylactic and therapeutic agents for enhancing and maintaining memory functions before the onset of memory impairments [1–3].

Traditional Chinese Medicine (TCM) has been frequently used for treating memory and cognitive deficits for thousands of years. The root of* Polygala tenuifolia*, a well-known medicinal plant in TCM, named as “Yuan Zhi,” has been commonly used as memory enhancer in China, and it is also included in some traditional prescriptions treating central nervous system disturbances, such as amnesia and dementia [4]. Recently, a number of studies have revealed that the root of* Polygala tenuifolia* could improve learning and memory function in animal models induced by scopolamine [5], KCN [6], *β*-amyloid peptide [7], stress, hypoxia [8], and accelerated senescence [9]. However, there is less research to verify the memory-enhancing effects of* Polygala tenuifolia* on the normal aged animals.

In the present study, we aim to investigate the effects of the crude extract of* Polygala tenuifolia* (EPT) on learning and memory impairments of the normal aged mice. Learning and memory parameters are evaluated by using Morris water maze and step-down passive avoidance tests. Additionally, potential mechanisms are also examined. Our results provide evidence that the improvement of learning and memory functions in normal aged mice by EPT may be via antagonizing oxidative damages as well as decreasing the activities of MAO and AChE.

## 2. Materials and Methods

### 2.1. Preparation of EPT

Air-dried roots of* Polygala tenuifolia* Willd. were purchased from the Company of Chinese Materia Medica in Beijing and identified by Professor Bengang Zhang from the Institute of Medicinal Plant Development, Chinese Academy of Medical Sciences (Beijing, China). A voucher specimen (no. 20090815) has been deposited in the Herbarium of the Institute. The dried roots (2 kg) were cut into small pieces and extracted exhaustively with boiling water for three times, each time for 1 h. The filtered liquid was loaded to a column of macroporous resin D101 (6 kg) (Cang Bon Adsorber Technology Co., Ltd., China) and eluted successively with water and ethanol-water (9 : 1, v/v), respectively. The ethanol-water liquid was evaporated in vacuo to yield a pale yellow residue (105 g), which is designated the crude extract of* Polygala tenuifolia* (EPT).

### 2.2. Drugs and Reagents

The test kits of malondialdehyde (MDA), superoxide dismutase (SOD), catalase (CAT), monoamine oxidase (MAO), and acetyl cholinesterase (AChE) were all purchased from Nanjing Jiancheng Bioengineering Institute (Nanjing, China). All the other reagents were of analytical grade from Sigma.

### 2.3. Animals

Female C57BL/6J mice (Sixty 15-month-old mice and twelve 3-month-old mice) were purchased from the Laboratory Animal Institute of Chinese Academy of Medical Science (Certification no. SCXK 2004-0006, Beijing, China). They were housed in groups of five animals per cage under a 12 : 12 h light-dark cycle at constant temperature (23°C ± 2°C) and humidity (50% ± 10%). The animals had free access to standard chow diet and sterilized drinking water in the SPF animal house. The 3-month-old mice were used as the normal control group. The 15-month-old mice were randomly assigned into six groups, including the normal control group, the aged control group, galantamine (3 mg/kg) group, and the various concentrations of EPT (50, 100, and 200 mg/kg, resp.) groups. The drug administration and behavioral assays were performed using a double-blind method. All animal experiments were conducted in compliance with the “Guide for the Care and Use of Laboratory Animals” of the Institute of Medicinal Plant Development, Chinese Academy of Medical Science and Peking Union Medical College.

### 2.4. Experimental Procedures

**Figure 1:**
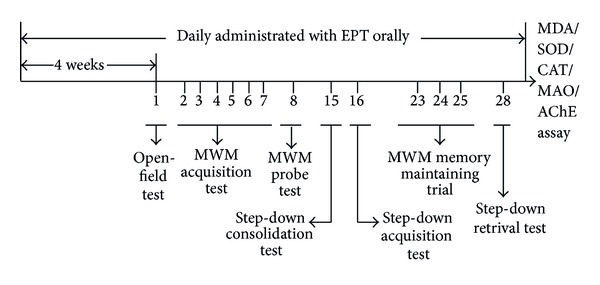
Experimental procedure.

After 2 days of habituation, mice received orally water (the normal control group and the aged control group), galantamine (3 mg/kg), or various concentrations of EPT (50, 100, and 200 mg/kg) for a period of 4 weeks before behavioral measurement was assessed. Locomotive activities of mice were tested in open-field on the first day of behavioral test. Morris water maze tests were carried out on day 2 to day 8 and day 23 to day 25; step-down passive avoidance tests were valued on day 15 to day 16 and day 28, respectively. Then, the mice were decapitated, and the brain tissues were dissected quickly on ice for detection of the content of MDA and the activities of SOD, CAT, MAO, and AChE. The procedure was presented in Figure 1.

### 2.5. Open-Field Test

The effect of EPT on mice locomotor activities was evaluated automatically using an open-field computer-aided controlling system as described in the literatures [10, 11]. The apparatus consists of four metal tanks (30 cm in diameter and 40 cm in height) with a video camera fixed at the top, and the apparatus was illuminated by a light source of 120 Lux on the ceiling. Experiments were performed in a quiet room; four mice were tested simultaneously. Thirty minutes after drug administration, each mouse was placed at the center of the metal tank and allowed to explore freely for 5 min. Then, the distance traveled by mouse was measured for 10 min, which was recorded to evaluate the locomotive activity of the mouse.

### 2.6. Morris Water Maze Test

#### 2.6.1. Apparatus

The apparatus (developed by Institute of Medicinal Plant Development, Chinese Academy of Medical Sciences, and Chinese Astronaut Center, Beijing, China) is a circular water pool (100 cm in diameter and 40 cm in height) with constant clues external to the maze for spatial orientation of the mice. The water was made opaque by adding black ink to prevent animals from seeing the submerged platform. The water temperature was kept at 24–26°C during the whole experiment. An invisible platform (6 cm in diameter and 15 cm in height) providing the only escape from water was placed 1.5 cm below the water surface. The pool was divided into four quadrants by a computerized tracking and image analyzer system. Two principal axes of the maze intersect perpendicularly to one another to create an imaginary “+.”The end of each line demarcates one of the four cardinal points: north (N), south (S), west (W), and east (E).

#### 2.6.2. Test Procedure

On days 2 to 8 of behavioral measurement, Morris water maze was used to assess the spatial reference memory consisting of an acquisition phase and a probe trial. Memory retention of the mice was tested on days 23 to 25.

In the acquisition phase, mice were placed in the pool containing platform to adapt to the environment before training. Then mice were subjected to two trials each day for 6 days to find the submerged platform that was located in the center of the SE quadrant of the pool and remained at the same position throughout the whole experiment. Two-day training of four trials contributed to a session. For each trial, the mouse was placed for 15 sec on the platform for learning; then, it was gently released into the pool facing the wall. Four different release points (NE, SE, SW, and NW) were varied randomly for each session. Animals were given a maximum of 60 sec to find the platform. If the mouse failed to find the platform within 60 sec, it was gently guided to the platform and stayed there for 10 sec, and its escape latency was recorded as 60 sec. If an animal found the platform within 60 sec, it was allowed to remain there for 10 sec and was then placed into a cage until next trial. After completion of daily training, the animals were returned to their cages for rest. Escape rate, escape latency, and swimming speed were collected to evaluate the ability of learning and memory function of mice.

On the 8th day of behavioral measurement, the spatial probe trials were tested. The platform was removed, and each mouse was placed into the water on the opposite side of the SE quadrant. They were allowed to swim freely for 120 s. The crossing numbers over the position at which the platform had been located, the swimming time, and the swimming distance spent in the target quadrant were recorded as measures for spatial memory.

Two weeks after Morris water maze tests, memory retention tests were given. Neither the platform nor the starting point was fixed; mice were released in the opposite quadrant. This training had been performed 2 times each day for 3 days. The average of two trials during a day was determined as escape latency for the purpose of evaluating memory retention abilities of mice.

### 2.7. Step-Down Test

Step-down passive avoidance tests were carried out in a chamber to evaluate the effects of EPT on learning and memory function of the aged mice. The floor of the chamber consisted of copper rods and a well-insulated platform made of rubber in one corner of the chamber. The animals were placed in the chamber for 3 min adaptation at the beginning of training. After adaptation, mice were placed on the floor and received an immediate mild electrical shock for 5 minutes (25 V). To avoid the shock, mice displayed an instinctive reaction to jump back onto the platform. The latency to step down on the grid with all four paws was measured. The time on the safe zone (on the platform) and time on the error zone (on the grid) were recorded within 4 min. The tests consisted of three phases of acquisition, consolidation, and retrieval, which were carried out on day 15, day 16, and day 28, respectively.

### 2.8. Preparation of Brain Tissue Samples and Biochemical Evaluation

After behavioral measurements, all the mice were sacrificed by decapitation; the brain tissues were quickly removed, washed with cold saline solution, followed by 50 mM Tris-HCl buffer (pH 7.4), and weighed. Then, it was placed into a glass bottle, labeled, and stored in a deep freezer (−25°C) until processing (maximum 10 hrs). The tissues were homogenized in four volumes of ice-cold Tris-HCl buffer (50 mM, pH 7.4) using a glass Teflon homogenizer (Elektrocrafts, Mumbai) for 2 min at 5000 rpm after cutting it into small pieces. The homogenate was then centrifuged (Remi, India) at 1000 rpm for 10 min to remove the debris. The clear upper supernatant fluid was extracted with an equal volume of ethanol and centrifuged at 17000 rpm for 30 min; the clear upper ethanol layer was taken and used for biochemical assay. All the preparations were performed at 4°C, and then MDA, SOD, CAT, MAO, and AChE were estimated using commercial kits according to the manufacturer's protocols.

#### 2.8.1. Determination of MDA Content in the Brain Tissue of Mice

The method of detecting MDA was based on a stable chromophoric product formed by reaction with thiobarbituric acid (TBA), which could be measured at the wavelength of 532 nm [12]. The calculation is MDA level (pmol/mg prot) = (absorbance of test tube − absorbance of blank tube)/(absorbance of standard tube − Absorbance of standard blank tube) × 10 nmol/mL × dilution multiple/protein level (mg prot/mL).

#### 2.8.2. Determination of SOD Activity in the Brain Tissue of Mice

The assay of total SOD was based on its ability to inhibit the oxidation by the xanthine-oxidase xanthine system [13]. The activities were quantified by measuring the amount of hydroxylamine nitrite produced by the oxidation of oxymin. The absorbance of the reaction product was measured at 550 nm. The calculation is SOD activity (U/mg prot) = (absorbance of control tube − absorbance of test tube)/absorbance of control tube/50% × dilution multiple/protein level (mg prot/mL).

#### 2.8.3. Determination of Catalase Activity in the Brain Tissue of Mice

The CAT activities were assessed by measuring the disappearance of hydrogen peroxide at 405 nm [14, 15]. All procedures complied with the manufacturer's instructions. One unit (U) of CAT corresponds to the amount of the enzyme that hydrolyses 1 mmol of hydrogen peroxide per minute at 25°C. The catalase activity was expressed as n moles of H_2_O_2_ metabolized/mg protein/h.

#### 2.8.4. Determination of MAO Activity in the Brain Tissue of Mice

The activity of MAO was based on the production of benzyl aldehyde from the reaction and its specific substrate, aniline hydrochloride [16]. Briefly, the protein concentrations were determined by the Lowry method using bovine serum albumin as a standard. The homogenate was then incubated (37°C, 30 min) in reaction buffer containing aniline hydrochloride. The absorbance of the reaction product was measured at 242 nm. One unit (U) of MAO activity was defined as the amount that increased the absorbance by 0.01 at 37°C. Therefore, MAO activity was expressed as U/mg protein.

#### 2.8.5. Determination of AChE Activity in the Brain Tissue of Mice

AChE activity was determined as described by Ellman et al. [17] with some modifications. In brief, 30 *μ*L of diluted homogenate was added to the reaction mixture, which contained 100 mM phosphate buffer (pH 8.0) and 1.0 mM 5,5′-dithiobis-2-nitrobenzoic acid (DTNB) in 2 mL, and incubated at 37°C for 6 min. Hydrolysis was monitored by the formation of the thiolate dianion of DTNB at 412 nm for 2-3 min with a spectrophotometer. AChE was calculated from the quotient between lymphocyte AChE activity and protein content. One unit (U) of enzyme activity was defined as 1 micromole of ACh hydrolyzed per hour and per mg of brain homogenate or per mL of blood (pH 8.0, 25°C).

### 2.9. Data Analysis

All tests were analyzed using the Statistical Package SPSS 16.0 (SPSS Inc., Illinois, Chicago, USA). Data were expressed as mean ± standard error of mean (S.E.M) and analyzed by one-way analysis of variance (ANOVA) followed by Dunnett's *t*-test. A value of *P* < 0.05 was considered statistically significant.

## 3. Results

### 3.1. Effect of EPT on Mouse Locomotive Activities in the Open-Field

**Figure 2:**
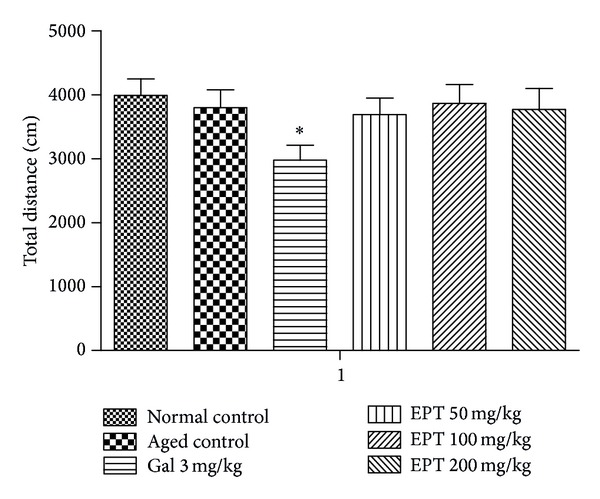
Effect of EPT on locomotive activities of mice. Data represent the mean ± S.E.M, *n* = 8–10 in each group. Gal represents galantamine 3 mg/kg group. The total distance traveled by mice was measured after repeated administrations of EPT (50, 100, and 200 mg/kg) for 4 weeks. *P < 0.05 compared with the aged control group.

As shown in Figure 2, no significant effect of EPT (50, 100, and 200 mg/kg) on mouse locomotive activities was observed in the open-field test. But galantamine 3 mg/kg reduced the total distance significantly compared with the aged control group (*P* < 0.05).

### 3.2. Effect of EPT on the Aged Mice in Morris Water Maze Test

#### 3.2.1. Acquisition Trials

**Figure 3:**
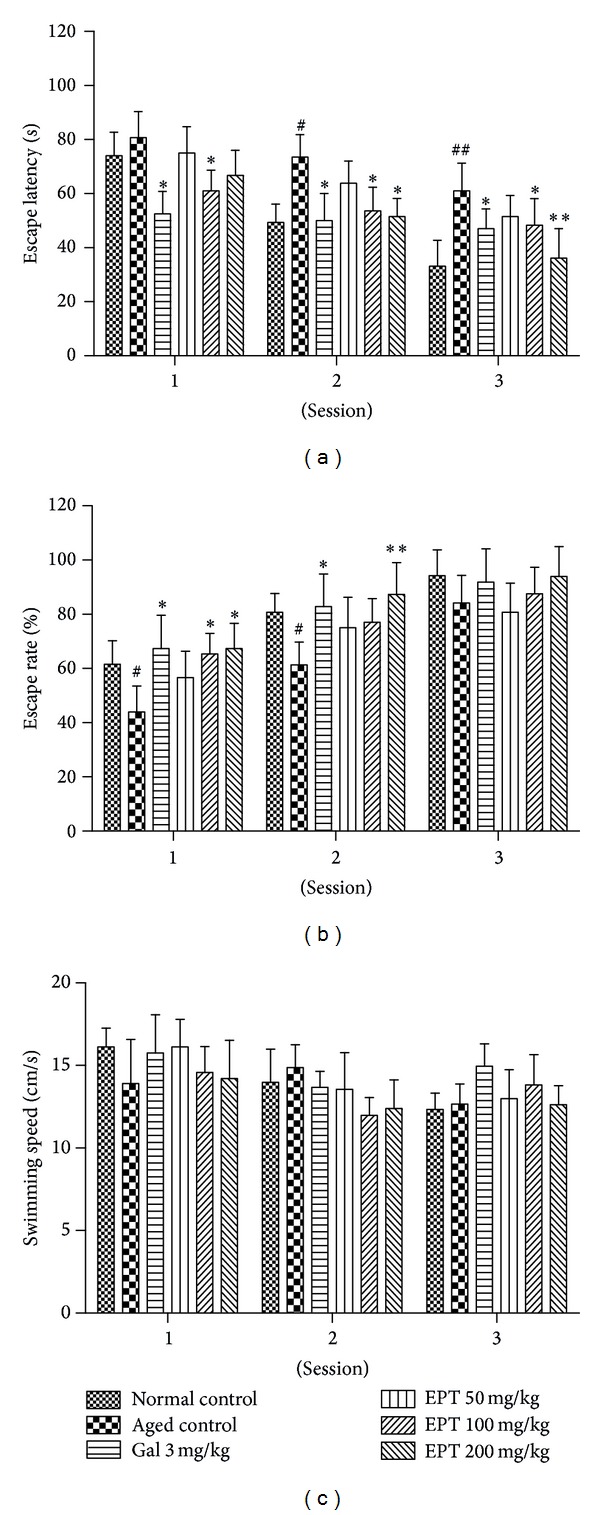
Effect of EPT on the acquisition phase in MWM tests of the aged mice. Training trials were carried out on day 2 to day 7 of behavioral tests, (a) escape latency, (b) escape rate, and (c) swimming speed. Gal 3 mg/kg represents galantamine 3 mg/kg group. Values represent mean ± S.E.M (*n* = 8–10 in each group). ^#^ *P* < 0.05 compared with the normal group; *P < 0.05 and **P < 0.01 compared with the aged control group.

As shown in Figures 3(a) and 3(b), the escape latency deceased and the escape rate increased over the course of training trials in all groups, which indicated that learning and memory functions of mice improved with the extension of training. Significant differences were observed between the normal control group (3-month mice) and the aged control group (15-month mice) at sessions 2 and 3 on the escape latency and at sessions 1 and 2 on escape rate in the training trials. The results indicated that 15-month mice took a longer time to locate the platform than 3-month mice did and 15-month mice had already undergone deterioration of the spatial memory ability. No significant differences appeared on the escape rate at session 3 when over 80% mice could find the platform. However, EPT could notably shorten the escape latency and raise the escape rate of the aged mice. Significant differences were observed in the EPT (200 and 100 mg/kg) treated groups as compared with the aged control group (*P* < 0.05 or *P* < 0.01). Similarly, the treatment with galantamine (3 mg/kg), a positive agent, showed little influence on the escape latency and escape rate. However, there were no significant differences on swimming speed in the whole training trials among all the groups (Figure 3(c)).

#### 3.2.2. Probe Trials

**Figure 4:**
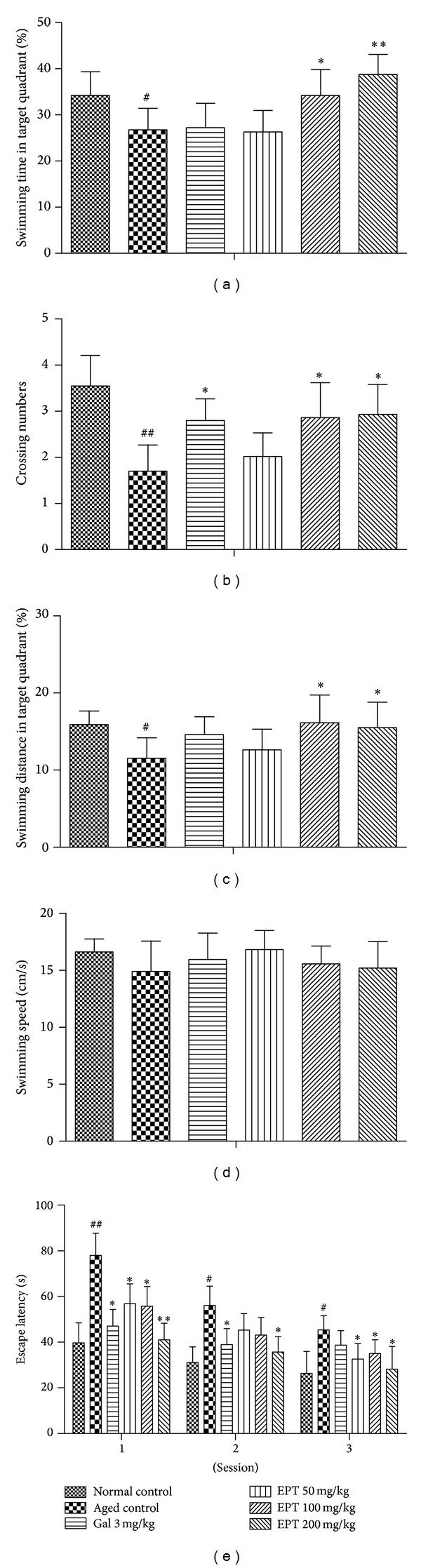
Effect of EPT on the probe and memory retention in MWM tests of the aged mice. The probe trial was performed 24 h after the acquisition test, (a) swimming time in target quadrant, (b) crossing numbers, (c) swimming distance in target quadrant, (d) swimming speed, and (e) escape latency on memory retention test. Gal 3 mg/kg represents galantamine 3 mg/kg group. Values represent mean ± S.E.M (*n* = 8–10 in each group). ^#^ *P* < 0.05 and ^##^ *P* < 0.01 compared with the normal control group; *P < 0.05 and **P < 0.01 compared with the aged control group.

As shown in Figure 4(b), the crossing numbers were significantly lower in the aged control mice than those in the normal control mice (*P* < 0.01). By contrast, EPT (100 and 200 mg/kg) and galantamine (3 mg/kg) increased the crossing numbers markedly (*P* < 0.05). Additionally, the shorter swimming time in the target quadrant in the aged mice was significantly reversed by EPT (100 and 200 mg/kg). (Figure 4(a), *P* < 0.05). A similar result was also observed for the swimming distance in the target quadrant (Figure 4(c), *P* < 0.05). Meanwhile, the swimming speed of all mice showed no differences in the probe trials (Figure 4(d)).

#### 3.2.3. Memory Retention Test

As shown in Figure 4(e), 15-month mice had much longer escape latency than 3-month mice in all the memory retention tests (*P* < 0.01 in session 1 and *P* < 0.05 in sessions 2 and 3, resp.). By contrast, the aged mice treated with different concentrations of EPT obviously cut down the escape latency as compared with the aged control mice. This indicated that EPT could promote memory retention ability of the aged mice to certain extent, and the significant differences were observed as shown in Figure 4(e) (*P* < 0.05 or *P* < 0.01).

### 3.3. Effect of EPT on the Aged Mice in Step-Down Task

**Figure 5:**
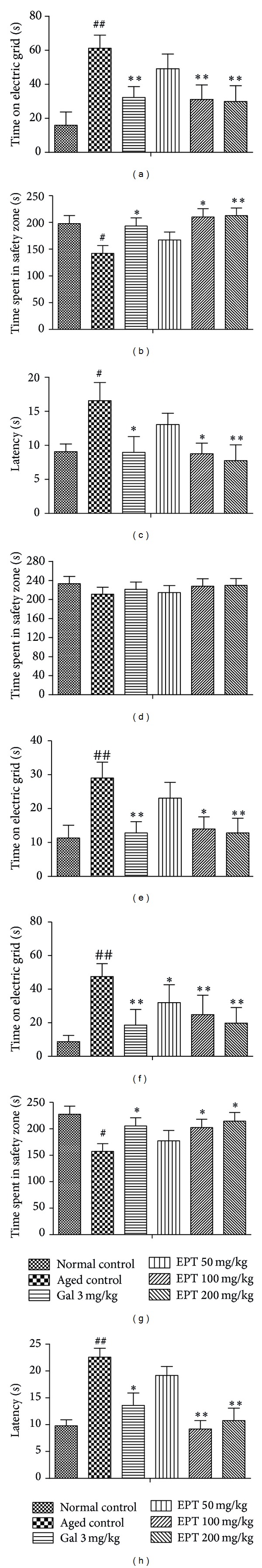
Effect of EPT on memory deficits of the aged mice in the step-down passive avoidance test. Figures 5(a)–5(c), 5(d) and 5(e), and 5(f)–5(h) represent the tests of acquisition, consolidation, and retrial, respectively. Figures 5(a), 5(e), and 5(f) show the time spent on electric grid; Figures 5(c) and 5(h) show latency; and Figures 5(b), 5(d), and 5(g) show the time spent in safety zone. Values represent mean ± S.E.M (*n* = 8–10 in each group). Gal 3 mg/kg represents galantamine 3 mg/kg group. ^#^ *P* < 0.05 and ^##^ *P* < 0.01, versus the normal control group; *P < 0.05 and **P < 0.01, versus the aged control group.

In the acquisition trials, the aged control mice revealed marked differences for the latency (Figure 5(c)), the time spent in the safe zone, and on the electric grid (Figures 5(b) and 5(a)) as compared with the normal control mice (*P* < 0.05 or *P* < 0.01). By contrast, EPT (100 and 200 mg/kg) and galantamine (3 mg/kg) could shorten the latency and the time spent on the electric grid and increase the time spent in the safety zone significantly (*P* < 0.01 or *P* < 0.05).

In the consolidation trials, the aged mice had longer time on the electric grid than the normal control mice (*P* < 0.01). By contrast, EPT (100 and 200 mg/kg) and galantamine (3 mg/kg) could decrease the time on the electric grid of the aged mice obviously as compared with that of the aged control mice (*P* < 0.01 or *P* < 0.05) (Figure 5(e)). However, no significant differences were observed in all groups on the time in safety zone (Figure 5(d)).

In the retrial tests, the aged control mice still displayed significant differences for the latency, the time spent in the safety zone, and the time spent on the electric grid as compared with the normal control mice. But by treatment with EPT and galantamine, learning and memory performances were manifestly improved, except low EPT group (50 mg/kg) (*P* < 0.01 or *P* < 0.05) (Figures 5(f)–5(h)).

### 3.4. Effect of EPT on MDA Levels and CAT and SOD Activities in the Brain Tissue of Mice

**Figure 6:**
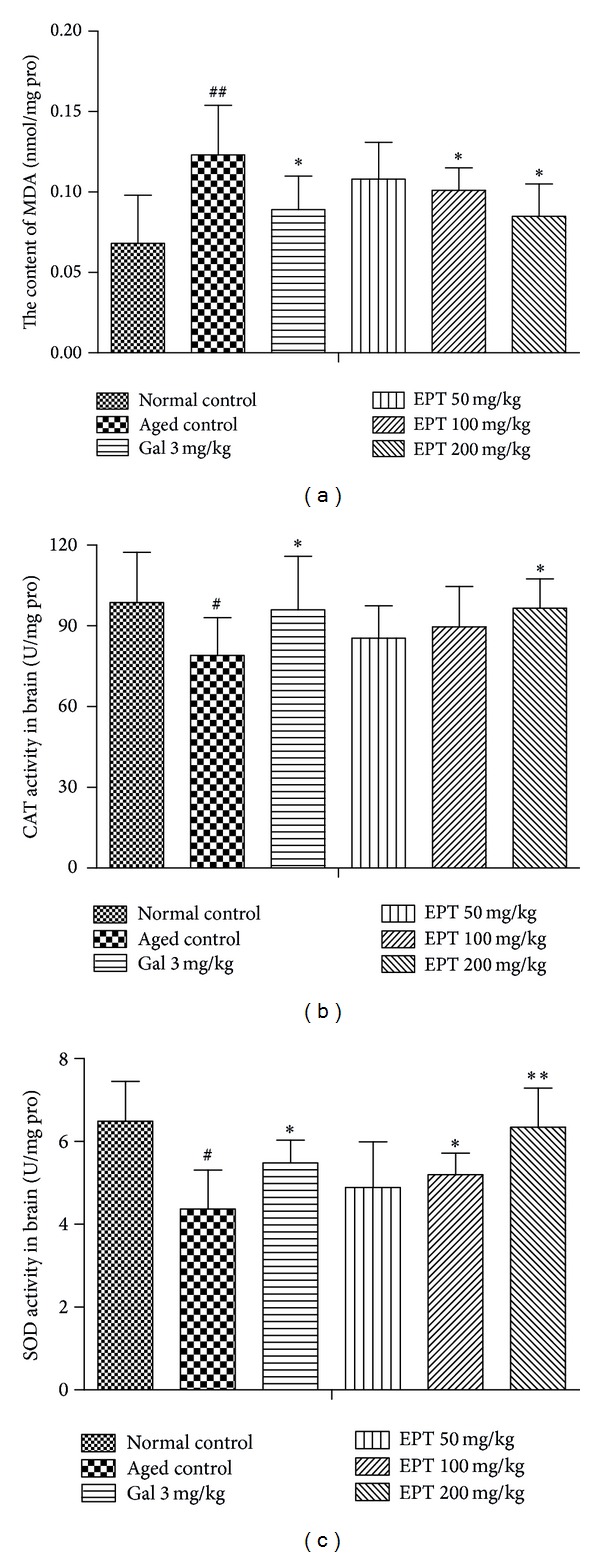
Effect of EPT on the changes of MDA levels and CAT and SOD activities in mice brain tissue. (a) MDA levels, (b) CAT activity, and (c) SOD activity. Values represent the mean ± S.E.M (*n* = 8–10 in each group). Gal represents galantamine 3 mg/kg group. ^#^ *P* < 0.05 and ^##^ *P* < 0.01, versus the normal control group; *P < 0.05 and **P < 0.01, versus the aged control group.

As shown in Figure 6(a), the MDA levels in the aged control mice were higher than those in the normal control mice (*P* < 0.01). Galantamine (3 mg/kg) and EPT (100 and 200 mg/kg) decreased the MDA levels significantly as compared with the aged control mice (*P* < 0.05). EPT (50 mg/kg) had the same tendency without statistical difference. As can been seen in Figures 6(b) and 6(c), both CAT and SOD activities of brain tissue in the aged control mice decreased significantly as compared with the normal control mice (*P* < 0.05). However, mice treated with EPT (200 mg/kg) and galantamine (3 mg/kg) showed high CAT and SOD activities as compared with the aged control mice (*P* < 0.05 or *P* < 0.01). There was a slight increase of both SOD and CAT activities in EPT (50, 100 mg/kg) groups, but no significant differences were observed compared with the aged control group except the group of EPT (100 mg/kg) for SOD activity (*P* < 0.05).

### 3.5. Effect of EPT on MAO and AChE Activities in the Brain Tissue of Mice

**Figure 7:**
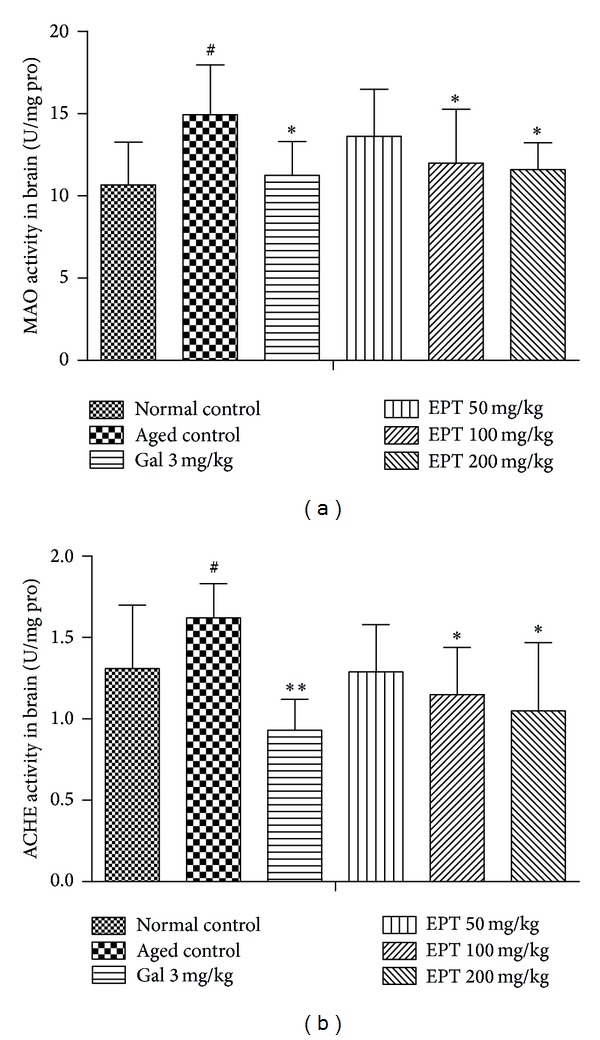
Effect of EPT on the changes of MAO (a) and AChE (b) activities in mice. Each value represents the mean ± S.E.M (*n* = 8–10 in each group). Gal represents galantamine 3 mg/kg group. ^#^ *P* < 0.05, versus the normal control group; *P < 0.05 and **P < 0.01, versus the aged control group.

As could be seen in Figures 7(a) and 7(b), the aged mice showed significant differences in MAO and AChE activities as compared with the normal control mice (*P* < 0.05). On the contrary, treated with EPT (100 and 200 mg/kg) and galantamine (3 mg/kg), the activities of MAO and AChE could markedly decrease (*P* < 0.05 or *P* < 0.01).

## 4. Discussion

Learning and memory abilities are important functions of the brain in humans and rodents. Aging in humans is associated with deterioration of cognitive performance, particularly, learning and memory abilities [18]. Aging animals have traditionally been used as a model of memory impairments [19]. Behavioral tests are one of the most reliable methods to investigate learning and memory abilities of animals. Generally, the animal's behavioral changes are detected by using Morris water maze and step-down and step-through tests [20–22]. The extract of* Polygala tenuifolia* (EPT) has been used as memory enhancer in Asia for thousands of years. Various animal models have demonstrated that EPT could improve brain functions [5–9]. In the present study, the memory-enhancing effects of EPT on the normal aged mice have been investigated by using Morris water maze and step-down passive avoidance tests.

Morris water maze is generally accepted as an indicator of spatial learning and reference memory. In the present study, spatial learning was assessed by repeated trials and evaluated by escape latency and escape rate; reference memory was determined by the preference of the platform area when the platform was absent and recorded by crossing numbers, swimming time, and swimming distance in the target quadrant; memory retention was measured by escape latency. In acquisition phase, different indexes revealed different sensitivities to evaluating learning and memory functions. When using the escape latency as an index, the learning and memory deterioration in aged control group appeared obviously different from the normal control group in all 3 sessions, but it could be counteracted partly by EPT (100 and 200 mg/kg) in session 2 and session 3 (Figure 3(a)). When using the escape rate as an index, EPT showed strong improving effects in all 3 sessions, but there was almost no difference among all groups in session 3 (Figure 3(b)). During the probe trials, EPT (100 and 200 mg/kg) significantly increased the swimming time and distance in the target quadrant (Figures 4(a) and 4(c)). This effect was not due to a different motor activity, for the mean swimming speed was not significantly different among all groups (Figure 4(d)). Moreover, the aged control group and EPT-treated groups showed comparable locomotor activity and emotional reactivity in the open-field test, which can also suggest that the amelioration of learning and memory in aged mice was not provoked by sensorimotor effects. In memory retention test, treatment with different concentrations of EPT on the aged mice obviously cut down the escape latency as compared with the aged control mice (Figure 4(e)).

Step-down passive avoidance task, which is a test of fear motivated inhibitory avoidance indicating nonspatial learning and memory [23], was performed to confirm the effects of EPT on another type of learning and memory ability of the aged mice. The results demonstrated that EPT could improve the memory impairment of the aged mice (Figure 5) and further proved the therapeutic effects of EPT. Moreover, latency, the time spent on the electric grid, and the time spent on the safety zone presented mutual support in acquisition and retrial trials. However, in consolidation trial, the time spent in the safety zone showed no obvious differences among all groups (Figure 5(d)), which might be related to the short-term memory because the consolidation test was carried out just 24 h after training trial. Taken together, EPT (100 and 200 mg/kg) improved the memory performances of the aged mice with multiple data in MWM and step-down tests.

It is well known that learning and memory deficits are accompanied by aging. The exact mechanisms responsible for the memory impairments with aging are still unclear, but evidence has accumulated that oxidative stress plays an important role [24–26]. Oxidative stress occurs when pro-oxidant and antioxidant levels become imbalanced. With aging, there is an increased production of reactive oxygen species (ROS) and diminished endogenous antioxidant enzyme levels, leading to an increased oxidizing cellular environment. Both SOD and CAT are the main endogenous antioxidant enzymes, playing an important role in the intracellular antioxidant defense in the brain. MDA, an important lipid peroxidation product, can be taken as an indicator for the state of oxidative damage of membranes under condition of oxidative stress. Two antioxidant markers SOD and CAT along with one oxidative stress marker MDA were measured in the current study. The results showed that the increasing MDA content and the decreasing activities of SOD and CAT in the aged mice could be partly reversed by EPT (100, 200 mg/kg) (Figure 6). These findings demonstrated that the memory enhancing effects of EPT on the aged mice may be via antioxidant system.

Aging is often accompanied by some alterations in the neurotransmitter systems such as acetylcholine and monoamine transmitters [27, 28]. And the transmission of these neurotransmitters in the brain has been long considered an important modulator of synaptic plasticity, memory consolidation, and other cognitive processes [29, 30]. Under normal condition, the metabolic controls, which are responsible for maintaining the levels of ACh and monoamine transmitters, are catalyzed by AChE and MAO, respectively [31, 32]. Our experimental data suggest that the EPT-mediated enhancement in spatial and non-spatial learning and memory abilities could be, at least partially, due to the decreasing activity of AChE and MAO in aged control mice (Figure 7), which were consistent with multiple behavioral tendencies.

In summary, administration of EPT not only ameliorated learning and memory deficits in the aged mice, but also generated antioxidant effect via the endogenous enzymatic system. Our results supported the fact that EPT possesses memory-enhancing effects in healthy [33] and elderly volunteers [34]. Although the mechanisms for the memory enhancing effects of EPT are still unclear, evidence is associated with complicated changes of neural network system and antiaging effects. Potential mechanisms which make EPT an effective agent for treating learning and memory disorders might be partially related to improved antioxidation and reduced activities of AChE and MAO. By combining analytical chemistry and chemogenomics [35] in the future, we aim to identify the memory-enhancing phytochemicals within EPT and elucidate their molecular mechanisms.
